# *Dorylaimida Mundi* (Nematoda)

**DOI:** 10.2478/jofnem-2022-0020

**Published:** 2022-06-29

**Authors:** Pablo Castillo

**Affiliations:** 1 Instituto de Agricultura Sostenible (IAS), Consejo Superior de investigaciones Científicas (CSIC), Córdoba, Spain

Dorylaims are one of the most diversified and abundant nematodes in natural and cultivated soils around the world, displaying high diversity in their morphology, behavior, and ecology. This group of nematodes has great importance as many of them can be considered biological/environmental indicators in soil ecological studies, while others are plant-parasites and virus vectors. As compiled in this book, Dorylaimida comprises 363 nominal genera and almost 3,500 nominal species. This heavy and interesting book encompasses four sections, including: (i) List of genera and species, with their records, comprising a catalog of the currently recognized taxa with their synonyms and reference citations; (ii) References; (iii) List of genera, i.e., an alphabetical list of taxa at the generic and subgeneric levels, together with their synonyms; and (iv) List of species, i.e., an alphabetical list of valid species names and their synonyms. These data provide a summarized yet complete picture of information on these plant-parasitic and soil nematodes worldwide. In my opinion, one of the greatest successes of this book is the compilation of a complete list of synonyms, including a comprehensive list of references in which the synonymy was proposed. For each genus, the book provides a list of the main references dealing with this taxon, indicating the keywords in brackets (viz. diagnosis, key for species, phylogeny, review, taxonomy, etc.). The main advantage of this strategy is that readers can easily perceive the complete history of each genus. This volume of 957 pages will contribute immensely toward a reader’s quest to find any taxonomical change of dorylaims until December 2020, but more important in my opinion, enable them to find the geographical distribution of genera and species that can be used in the future by those undertaking a study of this distribution, and realize the possibility to infer any potential global distribution pattern. In addition, an interesting information includes the annotation of an asterisk before a reference indicating that no taxonomic/ morphological information but only distribution data are cited, while a question mark suggests that the recorded taxon in the reference may be incorrect.

In summary, the author is to be congratulated for their initiative and achievement in the production of such an enjoyable book that is well written and comprises a valuable tool for those involved in the fields of basic and applied nematology, and biogeography and distribution (scientists, teachers, students).

